# Association between Fluid Balance and Treatment Outcome of Ibuprofen for Patent Ductus Arteriosus in Preterm Infants

**DOI:** 10.31083/j.rcm2403078

**Published:** 2023-03-03

**Authors:** Chang Liu, Yuan Shi

**Affiliations:** ^1^Department of Neonatology, Children’s Hospital of Chongqing Medical University, 400014 Chongqing, China; ^2^National Clinical Research Center for Child Health and Disorders, 400014 Chongqing, China; ^3^Ministry of Education Key Laboratory of Child Development and Disorders, 400014 Chongqing, China; ^4^Chongqing Key Laboratory of Pediatrics, 400014 Chongqing, China

**Keywords:** patent ductus arteriosus, fluid balance, ibuprofen, preterm infants

## Abstract

**Background:**

Excessive fluid intake is a predictor of the development of patent ductus arteriosus (PDA) in preterm infants. Previous studies have examined the relationship between fluid intake and outcomes following ibuprofen for PDA. However, there is a lack of data to determine whether fluid balance has an effect on ibuprofen treatment for PDA. Therefore, this study sought to determine the relationship between fluid balance and outcomes following treatment with ibuprofen for PDA.

**Methods:**

We conducted a retrospective study of 110 infants admitted to the Children’s Hospital of Chongqing Medical University between January 2017 and April 2022, who were treated with ibuprofen for hemodynamically significant PDA (hsPDA). We calculated the average fluid balance before and during the two courses of ibuprofen treatment and whether they were significantly associated with outcomes in hsPDA patients.

**Results:**

In the first course of ibuprofen treatment (FIT), responders had lower fluid balance before FIT compared to non-responders [median 31.82 (18.01, 39.66) vs 34.68 (25.31, 43.56) mL/kg/day; *p* = 0.049], while the fluid balance during FIT [median 40.61 (33.18, 63.06) vs 42.65 (30.02, 57.96) mL/kg/day;* p* = 0.703] did not differ between responders and non-responders. Fluid balance before the second course of ibuprofen treatment (SIT) (mean 41.58 ± 14.26 vs 35.74 ± 10.99 mL/kg/day; *p* = 0.322) and during SIT (mean 39.21 ± 12.65 vs 37.00 ± 21.38 mL/kg/day; *p* = 0.813) was not found to have a significant association with SIT outcome. Multivariate logistic regression analysis showed fluid balance before FIT was a predictor for FIT success [Odds ratio (OR): 0.967; 95% confidence interval (CI): 0.935–0.999; *p = *0.042]. Fluid balance within the first week of life had a greater association with the FIT outcome (OR: 0.967, 95% CI: 0.939–0.996, *p *= 0.027). Gestational diabetes mellitus and higher Apgar scores decreased the possibility of PDA closure after FIT.

**Conclusions:**

Lower fluid balance before FIT, especially within the first week of life appeared to be a predictor for closure of hsPDA after FIT in preterm infants.

## 1. Introduction

Patent ductus arteriosus (PDA), one of the most common cardiac conditions affecting preterm infants, is intimately associated with congestive heart failure, intraventricular hemorrhage (IVH), necrotizing enterocolitis (NEC), bronchopulmonary dysplasia (BPD) and death [1, 2, 3, 4]. However, due to the relatively high incidence of spontaneous closure of PDA in neonates and the controversies on the benefit-to-risk ratio of treatment, there is still no consensus on which PDAs to treat, when to treat, and how to treat [5, 6, 7, 8, 9]. The mainstay of therapy for preterm infants with hemodynamically significant PDA (hsPDA) is thought to be cyclooxygenase inhibitors (COXI), such as ibuprofen, indomethacin and paracetamol. COXI promotes ductal closure by inhibiting cyclooxygenase enzymes, thereby reducing the synthesis of prostaglandins that play a crucial role in maintaining the patency of the ductus. Compared with indomethacin, ibuprofen was reported to have similar efficacy but fewer side effects [5, 10, 11, 12, 13]. Recent meta-analyses found that paracetamol caused fewer adverse effects without significant differences in the PDA closure rates between ibuprofen and indomethacin [14, 15]. Nonetheless, the issue of whether dual-drug combinations may improve treatment results remains unclear, and further study in this area is required [16, 17].

Since Stevenson *et al*. [18] first reported that excessive fluid administration to preterm infants with respiratory distress syndrome (RDS) may be a predictor associated with the development of PDA complicating RDS, a growing body of research has studied the association between fluid intake and PDA, yielding conflicting results. Subsequent observational studies [19, 20, 21] and Bell’s [22] randomized controlled trial echoed the results from Stevenson’s research, suggesting that premature infants in the high fluid intake group after birth have an elevated risk of developing PDA, while some other randomized controlled trials came to a different conclusion [23, 24]. To address this dilemma, Bell *et al*. [25] conducted a Cochrane review which concluded that water restriction under the premise of avoiding substantial dehydration reduced the incidence of PDA without significantly raising the risk of adverse events. Based on this evidence, restricted fluid intake has been proposed to be one of the suggested non-pharmacological interventions for the management of PDA [5].

Considering the widely-acknowledged value of fluid restriction in the management of PDA, it is plausible to assume that fluid restriction probably enhances the response rate to COXI. There have been a few studies in an attempt to validate this inference, the majority of which observed no significant difference in fluid intake volume between responders and non-responders to COXI [3, 26, 27, 28, 29]. Only Ahamed *et al*. [30] provided evidence that fluid intake before indomethacin treatment would affect the incidence of PDA closure. However, there was considerable heterogeneity in these studies in terms of drug selection and the definition of mean fluid intake volume. In addition, these studies were restricted to the association between fluid intake volume and treatment outcome, but the crucial role that fluid balance played was not addressed.

Currently, there are no known studies evaluating whether fluid balance has a link with ibuprofen treatment and outcomes in PDA patients. Therefore this study was undertaken to determine the correlation between fluid balance and outcomes following treatment with ibuprofen for PDA in preterm infants.

## 2. Materials and Methods

### 2.1 Patients

We conducted a retrospective study at the Neonatal Intensive Care Unit (NICU) of Children’s Hospital of Chongqing Medical University, a tertiary hospital in southwest China. We included preterm infants gestational age (GA) <32 weeks admitted from January 2017 to April 2022, who satisfied the inclusion and exclusion criteria described below.

Inclusion criteria for preterm infants in this study were as follows: GA <32 weeks, admitted to the NICU within 24 hours after birth, diagnosed as hsPDA according to our diagnostic criteria below, and at least one course of COXI treatment. The exclusion criteria were incomplete data, severe congenital anomalies, and an incomplete course of COXI (<3 doses) because of severe medical complications.

### 2.2 Diagnostic Criteria for hsPDA

A diagnosis of hsPDA was made by both echocardiography and clinical presentation. The echocardiographic diagnostic criteria were as follows: ductal size ≥1.5 mm and significant blood flow through the ductus. The clinical diagnostic criteria were met if the infant with a significant cardiac murmur at the left sternal border exhibited one of the following manifestations: dyspnea, tachypnea, apnea, and hypotension.

### 2.3 HsPDA Treatment

Echocardiographic examinations were performed in all infants with cardiac murmurs upon admission to the NICU. If an infant was found to have hsPDA, consecutive echocardiographic examinations were conducted and pharmacological or surgical treatment was initiated to close the hsPDA. One full course of intravenous or oral ibuprofen treatment was administered in three doses: 10 mg/kg for the first day, followed by two additional doses of 5 mg/kg on days two and three. Ibuprofen was given to infants without contraindications. If complications occurred during treatment, the medication was immediately discontinued. The contraindications and complications of ibuprofen treatment include severe pulmonary hypertension, uncontrolled septicemia, ductus-dependent congenital heart disease, renal insufficiency, intracranial hemorrhage, and gastrointestinal bleeding. Echocardiography was repeated within 3 days after the last dose of a course of ibuprofen to assess the treatment response. A successful response to ibuprofen treatment was echocardiographically defined as a ductal size <1.5 mm or insignificant ductal shunt flow. In the absence of these outcomes, the case was defined as a treatment failure. Further pharmacological treatment was administered at the same dose for infants who failed to respond to the first course or whose PDA reopened. Surgical ligation was also considered, especially in infants with repeated failed attempts for ibuprofen closure or those with contraindication to ibuprofen.

### 2.4 Data Collection

The following data was recorded for each infant: GA, birth weight (BW), sex, small for gestational age (SGA), multiple gestation, type of delivery, Apgar scores at 1, 5, 10 min, antenatal steroid, premature rupture of membranes (PROM), maternal hypertension, gestational diabetes mellitus, sepsis, RDS, BPD, death, surfactant administration, mechanical ventilation administration, blood transfusion administration, furosemide administration, hospitalization duration, arterial blood gas and blood routine indexes within 24 hours after birth, ductus diameter before treatment and postnatal age at two doses of ibuprofen. In addition, fluid-related data including intravenous intake volume, oral intake volume, and output volume were systematically collected.

RDS was diagnosed by clinical signs and symptoms, including respiratory distress, tachypnea, nasal flaring, groan, and cyanosis, appearing within 24 hours of birth, as well as an effective response to pulmonary surfactant and/or lung recruitment strategies. Other criteria included typical radiographic features such as a grainy shadow, air bronchogram, or white lungs [31]. BPD was diagnosed and graded following the 2001 National Institute of Child Health and Human Development (NICHD) consensus [32] and sepsis was diagnosed according to the Chinese Expert Consensus (version 2019) [33].

### 2.5 Statistical Analysis

We calculated fluid balance by subtracting the daily fluid output from the daily fluid intake. An intravenous or oral fluid of any type was considered intake. Output included urine and fluid loss from drains and tubes. Insensible water loss was not considered. Data regarding fluid intake and output was routinely recorded every day by NICU nurses. Statistical analysis was performed using SPSS 25.0 software (IBM, Chicago, IL, USA). Data normality was tested by the Shapiro-Wilk test. Continuous variables were evaluated using the Student’s *t* test or Mann-Whitney U test according to the normality of distribution. Categorical variables were tested by chi-square or Fisher’s exact test. Continuous variables were presented as mean ± SD (standard deviation) or median [quartile (Q) 25–Q75], and categorical variables were shown as frequency (percentages). Multivariable logistic regression was used to assess the independent contribution of potential factors to the outcome. The Odds ratio (OR) and 95% confidence interval (CI) were calculated. Spearman test was carried out to examine how closely the fluid balance correlates with changes in weight. The receiver-operating characteristic (ROC) curve analysis was used to determine the predictive accuracy of potentially significant factors in identifying responders and non-responders to ibuprofen. A two-tailed *p-*value of <0.05 was considered statistically significant.

## 3. Results

### 3.1 Characteristics of the Study Population

**Fig. 1.**
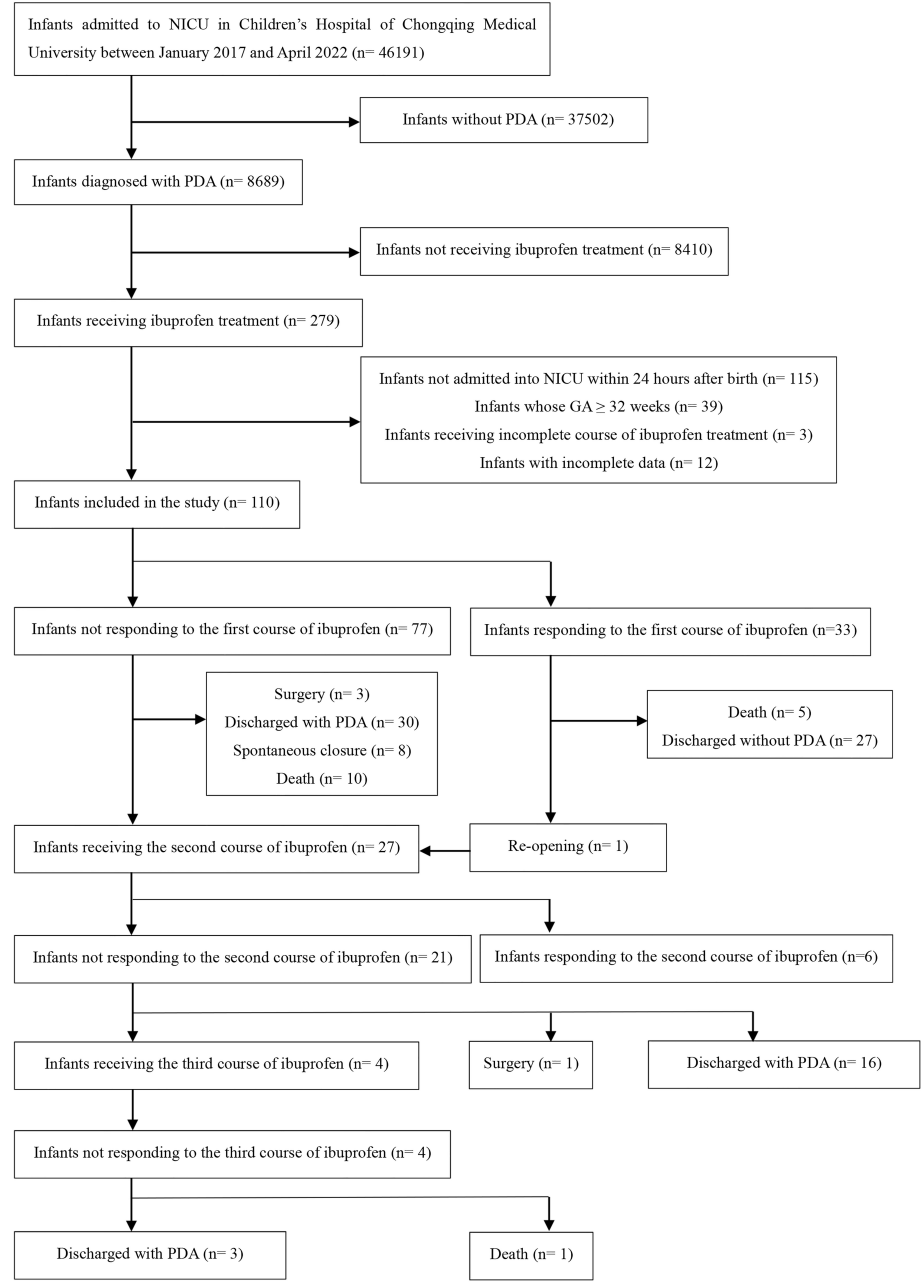
**Study flow chart**. Abbreviations: NICU, neonatal intensive care unit; PDA, patent ductus arteriosus; GA, gestational age.

During the study period, 46,191 infants were admitted to our NICU, of which 110 were included in the study after exclusion criteria were applied (Fig. 1). Tables 1,2 displayed the cohort’s baseline characteristics. The first course of ibuprofen treatment (FIT) and the second course of ibuprofen treatment (SIT) were successful in 30.0% (33/110) and 22.2% (6/27) of the infants, respectively.

**Table 1.** **Comparisons of baseline characteristics between responders and non-responders to the first course of ibuprofen treatment**.

Factor	Responders (n = 33)	Non-responders (n = 77)	*p*-value
Male	19 (57.6%)	43 (55.8%)	0.867
GA (weeks)	29 (27, 30)	28 (27, 30)	0.478
BW (grams)	1120 (860, 1330)	1140 (1000, 1445)	0.593
Cesarean section	25 (75.8%)	46 (59.7%)	0.108
Multiple birth	15 (45.5%)	28 (36.4%)	0.371
PROM	14 (42.4%)	27 (35.1%)	0.464
Maternal hypertention	5 (15.2%)	13 (16.9%)	0.822
Gestational diabetes mellitus	5 (15.2%)	31 (40.3%)	0.010*
Antenatal steroids	21 (63.6%)	59 (76.6%)	0.161
SGA	4 (12.1%)	2 (2.6%)	0.119
Apgar 1 min	6 (3, 8)	7 (6, 8)	0.009*
Apgar 5 min	8 (7, 9)	9 (8, 10)	0.004*
Apgar 10 min	9 (7.5, 10)	9 (8, 10)	0.038*
RDS	31 (93.9%)	74 (96.1%)	1.000
Sepsis	25 (75.8%)	61 (79.2%)	0.687
BPD	26 (78.8%)	62 (80.5%)	0.835
Death	5 (15.2%)	11 (14.3%)	1.000
Surfactant	30 (90.9%)	73 (94.8%)	0.733
Mechanical ventilation	30 (90.9%)	68 (88.3%)	0.947
Blood transfusion	29 (87.9%)	69 (89.6%)	1.000
Furosemide	27 (81.8%)	61 (79.2%)	0.755
Hospitalization length (days)	59 (47, 79.5)	70 (54, 89)	0.117
Platelet (×${\mathrm{10}}^{9}$/L)	204 (146.5, 237)	196 (148.5, 244)	0.749
Hemoglobin (g/L)	173.88 ± 36.92	174.58 ± 28.28	0.914
MPV (fL)	10.80 ± 0.74	10.61 ± 0.83	0.256
PDW	12.3 (11.45, 13.7)	12.3 (11.35, 13.45)	0.802
Lymphocyte (×${\mathrm{10}}^{9}$/ L)	2.58 (1.895, 3.965)	3.13 (2.05, 4.12)	0.311
Neutrophils (×${\mathrm{10}}^{9}$/ L)	4.53 (3.525, 7.49)	6.17 (3.67, 9.535)	0.033*
NLR	1.93 (1.14, 2.90)	2.31 (1.57, 4.06)	0.065
Lactate (mmol/L)	3.1 (1.75, 5.2)	2.4 (1.4, 3.6)	0.095
pH	7.3 (7.205, 7.4)	7.3 (7.205, 7.38)	0.501
Age at treatment (days)	10 (7, 19.5)	16 (11, 22.5)	0.052
Diameter of PDA (mm)	3.2 (2.7, 3.85)	3.4 (2.9, 4.1)	0.542
Intravenous ibuprofen	0 (0%)	5 (6.5%)	0.318

Abbreviations: GA, gestational age; BW, birth weight; PROM, premature rupture of membranes; SGA, small for gestational age; RDS, respiratory distress syndrome; BPD, bronchopulmonary dysplasia; MPV, mean platelet volume; PDW, platelet distribution width; NLR, neutrophil-to-lymphocyte ratio; PDA, patent ductus arteriosus. *p < 0.05.

**Table 2.** **Comparisons of baseline characteristics between responders and non-responders to the second course of ibuprofen treatment**.

Factor	Responders (n = 6)	Non-responders (n = 21)	*p*-value
Male	2 (33.3%)	14 (66.7%)	0.320
GA (weeks)	29.33 ± 1.86	28.67 ± 1.49	0.369
BW (grams)	1446.67 ± 483.76	1225.71 ± 330.14	0.204
Cesarean section	4 (66.7%)	12 (57.1%)	1.000
Multiple birth	3 (50.0%)	11 (52.4%)	1.000
PROM	2 (33.3%)	10 (47.6%)	0.877
Maternal hypertention	0 (0.0%)	4 (19.0%)	0.545
Gestational diabetes mellitus	2 (33.3%)	12 (57.1%)	0.571
Antenatal steroids	5 (83.3%)	17 (81.0%)	1.000
SGA	0 (0.0%)	0 (0.0%)	NA
Apgar 1 min	7.33 ± 2.25	6.95 ± 1.53	0.633
Apgar 5 min	10 (9, 10)	9 (8, 9.5)	0.122
Apgar 10 min	9 (10, 10)	9 (8, 9.5)	0.212
RDS	6 (100.0%)	20 (95.2%)	1.000
Sepsis	3 (50.0%)	18 (85.7%)	0.194
BPD	5 (83.3%)	18 (85.7%)	1.000
Death	0 (0.0%)	2 (9.5%)	1.000
Surfactant	5 (83.3%)	20 (95.2%)	0.402
Mechanical ventilation	5 (83.3%)	19 (90.5%)	0.545
Blood transfusion	6 (100.0%)	20 (95.2%)	1.000
Furosemide	3 (50.0%)	17 (81.0%)	0.318
Hospitalization length (days)	54 (41.5, 104.5)	75 (62, 94)	0.080
Platelet (×${\mathrm{10}}^{9}$/L)	205.83 ± 58.10	230.15 ± 160.94	0.723
Hemoglobin (g/L)	194.67 ± 19.29	167.95 ± 28.81	0.045*
MPV (fL)	10.50 ± 0.59	10.39 ± 1.11	0.812
PDW	11.88 ± 0.94	11.78 ± 2.45	0.921
Lymphocyte (×${\mathrm{10}}^{9}$/L)	3.52 (3.24, 3.70)	3.13 (2.44, 3.41)	0.808
Neutrophils (×${\mathrm{10}}^{9}$/L)	12.97 (7.31, 17.83)	5.77 (3.98, 9.01)	0.248
NLR	4.00 (2.65, 5.07)	2.20 (1.30, 3.00)	0.114
Lactate (mmol/L)	1.3 (1.2, 1.4)	2.5 (1.45, 2.75)	0.037*
pH	7.24 ± 0.09	7.32 ± 0.11	0.160
Age at treatment (days)	21 (15, 21)	28 (19, 35)	0.144
Diameter of PDA (mm)	2.8 (2.5, 3.6)	3.5 (3.15, 4.0)	0.090
Intravenous ibuprofen	0 (0.0%)	0 (0.0%)	NA

Abbreviations: GA, gestational age; BW, birth weight; PROM, premature rupture of membranes; SGA, small for gestational age; RDS, respiratory distress syndrome; BPD, bronchopulmonary dysplasia; MPV, mean platelet volume; PDW, platelet distribution width; NLR, neutrophil-to-lymphocyte ratio; PDA, patent ductus arteriosus; NA, not applicable. *p < 0.05.

Most of the baseline characteristics, including GA, BW, and sex in both FIT and SIT, were not significantly different between responders and non-responders (*p >* 0.05), but several baseline variables were statistically different between the two groups. In FIT, we observed that exposure to gestational diabetes mellitus (15.2% vs 40.3%; *p* = 0.010) and higher neutrophil counts [4.53 (3.525, 7.49) vs 6.17 (3.67, 9.535); *p* = 0.033] increased the probability of treatment failure. In addition, infants with lower Apgar scores at 1 min [6 (3, 8) vs 7 (6, 8);* p* = 0.009] were more likely to respond to FIT. Similar results were also obtained for Apgar scores at 5 and 10 min (Table 1). Responders showed higher hemoglobin levels (194.67 ± 19.29 vs 167.95 ± 28.81 g/L; *p* = 0.045) and lower lactate levels [1.3 (1.2, 1.4) vs 2.5 (1.45, 2.75); *p* = 0.037] in SIT compared to non-responders (Table 2).

### 3.2 Accuracy of the Measured Fluid Balance

To verify our fluid balance measurement, we assessed how closely measured fluid balance corresponds with weight changes by conducting a linear analysis between mean daily weight changes and mean daily fluid balance of all included infants. The results showed that measured fluid balance has a marginally significant correlation with weight changes (r = 0.195;* p* = 0.113), indicating that the accuracy of the measured fluid balance in our study was barely satisfactory.

### 3.3 Associations between Fluid-Related Factors and Treatment Outcome

**Fig. 2.**
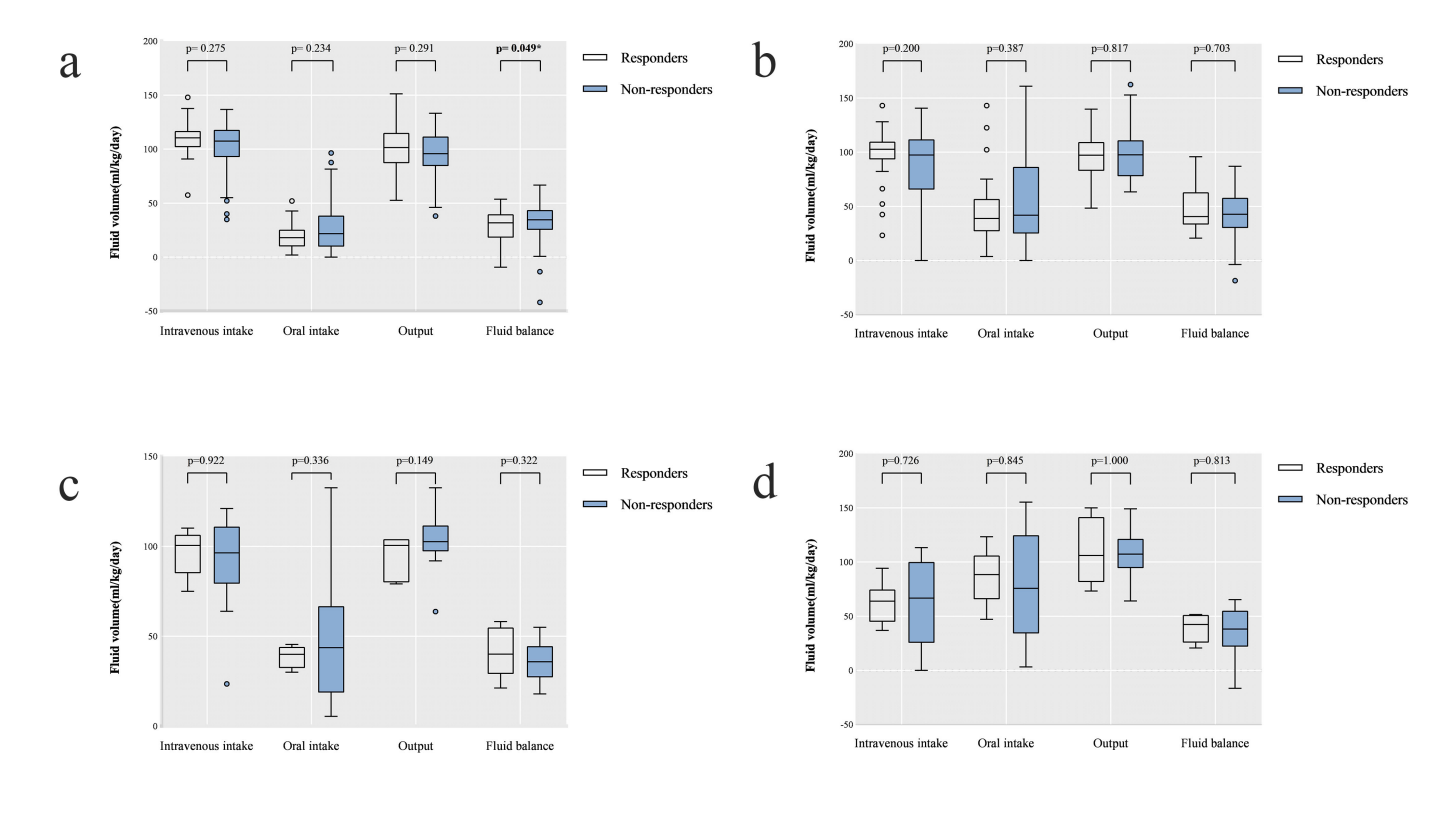
**Box plots of intravenous intake, oral intake, output and fluid balance volume of responders and non-responders in two courses of ibuprofen treatment**. (a) Description of these fluid-related variables before FIT. (b) Description of these fluid-related variables during FIT. (c) Description of these fluid-related variables before SIT. (d) Description of these fluid-related variables during SIT. Abbreviations: FIT, the first course of ibuprofen treatment; SIT, the second course of ibuprofen treatment. *p < 0.05.

According to univariate analysis, fluid balance before FIT was slightly significantly lower in infants successfully responding to FIT compared to those with failed closure of hsPDA [31.82 (18.01, 39.66) vs 34.68 (25.31, 43.56); *p* = 0.049; Table 3, Fig. 2A]. However, no other fluid-related pre- or intra-treatment factors were shown to be statistically correlated with treatment results in two courses of ibuprofen treatment (Tables 3,4 and Fig. 2B–D).

**Table 3.** **Comparisons of fluid-related data between responders and non-responders to the first course of ibuprofen treatment**.

Factor	Responders (n = 33)	Non-responders (n = 77)	*p*-value
Before the first course of ibuprofen treatment (mL/kg/day)			
Total fluid balance	31.82 (18.01, 39.66)	34.68 (25.31, 43.56)	0.049*
Intravenous intake	110.40 (101.85, 116.86)	107.52 (92.65, 117.86)	0.275
Oral intake	18.02 (9.82, 25.40)	21.72 (9.66, 38.42)	0.234
Output	100.61 ± 20.10	96.18 ± 20.03	0.291
During the first course of ibuprofen treatment (mL/kg/day)			
Total fluid balance	40.61 (33.18, 63.06)	42.65 (30.02, 57.96)	0.703
Intravenous intake	102.61 (93.39, 109.79)	97.47 (65.44, 111.83)	0.200
Oral intake	38.79 (26.96, 56.76)	41.87 (24.96, 86.45)	0.387
Output	97.28 (82.77, 109.32)	97.65 (77.83, 110.05)	0.817

*p < 0.05.

**Table 4.** **Comparisons of fluid-related data between responders and non-responders to the second course of ibuprofen treatment**.

Factor	Responders (n = 6)	Non-responders (n = 21)	*p*-value
Before the second course of ibuprofen treatment (mL/kg/day)			
Total fluid balance	41.58 ± 14.26	35.74 ± 10.99	0.322
Intravenous intake	100.50 (85.09, 106.43)	96.40 (79.26, 111.00)	0.922
Oral intake	38.56 ± 6.23	45.97 ± 32.13	0.336
Output	93.68 ± 12.65	103.94 ± 14.04	0.149
During the second course of ibuprofen treatment (mL/kg/day)			
Total fluid balance	39.21 ± 12.65	37.00 ± 21.38	0.813
Intravenous intake	63.48 (42.15, 66.17)	66.77 (25.46, 99.99)	0.726
Oral intake	86.55 ± 26.46	80.84 ± 48.92	0.845
Output	112.65 (78.78, 144.36)	107.32 (94.43, 121.47)	1.000

### 3.4 ROC Curve and Multivariable Regression Analysis

**Fig. 3.**
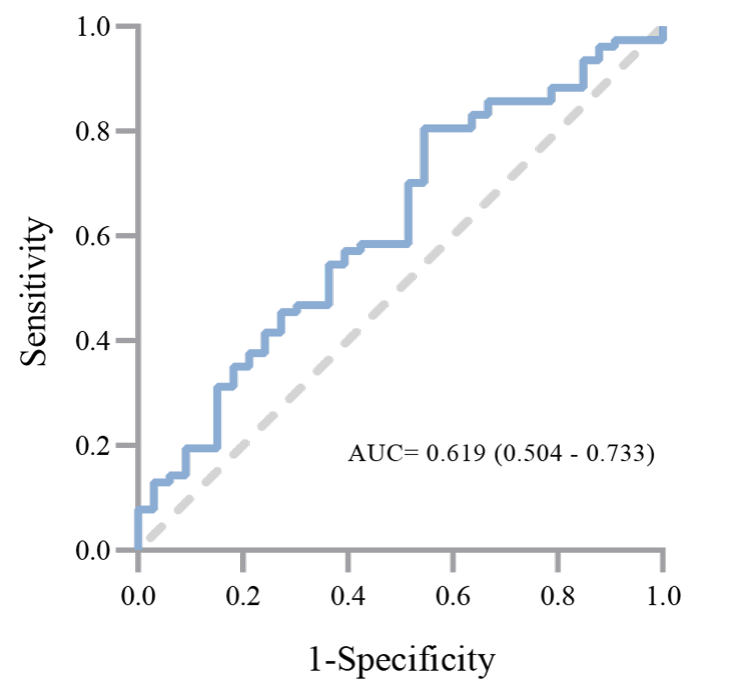
**ROC curve describing the fluid balance before FIT predicting FIT success**. AUC was 0.619 and a CI of 0.504–0.733. Abbreviations: ROC, receiver-operating characteristic; FIT, the first course of ibuprofen treatment; AUC, area under the curve; CI, confidence interval.

We performed a ROC curve analysis, which demonstrated a significant relationship between fluid balance prior to FIT and FIT success (Fig. 3). A fluid balance volume of 23.90 mL/kg/day (sensitivity: 0.805, specificity: 0.455) was calculated as the cutoff with an area under the curve (AUC) of 0.619 and a 95% CI of 0.504–0.733.

The findings of the multivariable logistic regression analysis were shown in Table 5. Because Apgar scores at 5 min and 10 min are closely associated with Apgar scores at 1 min, we eliminated them from the regression model due to collinearity. Even though the GA did not differ between responders and non-responders in FIT and SIT, we included it as a variable in the multivariable logistic regression analysis.

**Table 5.** **Multivariate regression analysis of successful closure of PDA by FIT and SIT**.

Factor in FIT	OR	95% CI	*p*-value	Factor in SIT	OR	95% CI	*p*-value
Gestational diabetes mellitus	0.200	0.062–0.649	0.007*	Hemoglobin (g/L)	1.057	0.987–1.131	0.114
Apgar 1 min	0.761	0.615–0.941	0.012*	Lactate (mmol/L)	0.149	0.015–1.453	0.101
Neutrophils (×${\mathrm{10}}^{9}$/L)	0.897	0.803–1.001	0.052	GA	0.717	0.269–1.914	0.507
Total fluid balance before ibuprofen treatment (mL/kg/day)	0.967	0.935–0.999	0.042*				
GA	0.933	0.694–1.254	0.646				

Logistic regression successful closure of PDA by FIT was performed adjusted for GA, gestational diabetes mellitus, Apgar 1 min, and neutrophils. Logistic regression successful closure of PDA by SIT was performed adjusted for GA, Hemoglobin, and Lactate. Abbreviations: PDA, patent ductus arteriosus; FIT, the first course of ibuprofen treatment; SIT, the second course of ibuprofen treatment; GA, gestational age; OR, odds ratio; CI, confidence interval. *p < 0.05.

The logistic regression showed that the likelihood of effectively closing the hsPDA in the FIT was improved by a reduced fluid balance volume before FIT (OR: 0.967; 95% CI: 0.935–0.999; *p* = 0.042), less frequent exposure to gestational diabetes mellitus (OR: 0.200; 95% CI: 0.062–0.649; *p* = 0.007), and lower Apgar scores at 1 min (OR: 0.761; 95% CI: 0.615–0.941; *p* = 0.012), in addition to which, lower neutrophil counts also indicated a favorable tendency for the FIT to successfully close hsPDA, however, no statistical significance was attained (*p *= 0.052). After the multivariable logistic regression, no potential factors could predict treatment outcome in SIT (Table 5).

Besides, further investigations were performed and we found that fluid balance within the first week of life had a greater association with the outcome of FIT (OR: 0.967, 95% CI: 0.939–0.996, *p *= 0.027), while the average fluid balance from day 8 after birth to the start of FIT had no substantial effect on FIT outcome (Table 6).

**Table 6.** **Multivariate regression analysis of successful closure of PDA by the first course of ibuprofen treatment**.

Factor	OR	95% CI	*p*-value
Fluid balance within the first week of life	0.967	0.939–0.996	0.027*
Fluid balance from day 8 after birth to the start of FIT	0.998	0.969–1.028	0.904

Logistic regressions were separately performed, adjusted for GA, gestational diabetes mellitus, Apgar 1 min, and neutrophils. Abbreviations: PDA, patent ductus arteriosus; FIT, the first course of ibuprofen treatment; GA, gestational age; OR, odds ratio; CI, confidence interval. *p < 0.05.

## 4. Discussion

In this study, we identified a decreased risk of FIT failure among infants with lower fluid before FIT, especially within the first week after birth. We also observed that gestational diabetes mellitus, higher Apgar scores, and higher neutrophil counts reduced the possibility of successfully responding to FIT. However, the statistically significant association between higher neutrophil counts and non-response to FIT was no longer present after multivariable regression analysis was performed. There was no independently significant correlation between any of the variables and the outcome of SIT and fluid balance after the first week, nor was it significantly related to FIT outcome.

Our study found it was fluid balance, not fluid intake before FIT, which was significantly associated with the FIT outcome. The overall fluid balance, which is simultaneously affected by intake volume and output volume, is a more integrated factor than intake volume and more precisely depicts the overall fluid load. In their randomized controlled trial, Bell *et al*. [22] proposed that the association between PDA development and fluid overload was probably attributed to water retention and a disturbance in hemodynamic balance when liberal amounts of fluid are given, resulting in larger systemic-to-pulmonary shunting through the ductus arteriosus. However, De Buyst *et al*. [34] concluded that fluid restriction did not reduce pulmonary circulation overload in their prospective observational study. Additionally, this association is also postulated to be triggered by an increase in prostaglandin E2 levels resulting from excessive fluid intake [35, 36, 37].

In the initial analysis, we noticed that only the fluid balance before FIT had a significant association with FIT outcome, while there was no statistically significant association between treatment outcome and fluid balance before and during SIT, and during the FIT. Potential explanations were the small sample size of infants treated with SIT and the short duration of the treatment period, only three days, which might not be sufficient to have a strong impact on treatment outcomes. In addition, given that the immediate postnatal days are an important period of physiological adaptation for the newborns, with significant hemodynamic changes and a greater susceptibility to hemodynamic instability [38, 39], the inconsistent results could possibly be also interpreted by the hypothesis that only fluid balance within a limited period of time after birth had a significant effect on the outcome of FIT, and the longer the time after birth, the less impact fluid balance had on FIT outcome. Accordingly, we did further investigations on the relationship between fluid balance within the first week of life, fluid balance from day 8 after birth to start of FIT and FIT outcome, respectively. The results of multivariate regressions were compatible with our assumption and showed that only fluid balance within the first week had a significant association with the outcome of FIT (*p* = 0.027) and was a more robust predictor for the outcome of FIT than the overall fluid balance before FIT (*p* = 0.042).

From the results of our study and the conclusions of the previous literature [18, 19, 20, 21, 22, 25, 40, 41], fluid restriction is extremely important for infants after birth. However, sometimes fluid restriction has negative repercussions such as nutritional deficiency, dehydration, hypotension, and impaired endorgan perfusion [42, 43]. According to the Canadian Pediatric Society, “aggressive” fluid restriction (120 mL/kg/day) may be potentially hazardous in preterm newborns with hsPDA [44]. Diuretic medications are another option to restrict fluid balance. But a neonatal rodent experimental model demonstrated that furosemide might delay ductal closure and might also lead to dilation of the constricting PDA. Furosemide could promote renal production of prostaglandin E2, a potent ductus arteriosus dilator. Premature infants may be at an increased risk for suffering from electrolyte disturbances, thrombocytopenia, acute kidney injury, nephrocalcinosis, ototoxic sensorineural hearing loss, and direct hyperbilirubinemia due to the use of furosemide [45, 46]. Because of these serious side effects, furosemide is not generally accepted as a standard part of conservative management of the hsPDA [47, 48]. Some pediatric cardiology and neonatology groups have begun recommending chlorothiazide as a first-line for diuretic in hsPDA [49]. Since restricting fluid intake and the use of diuretics pose a higher risk of complications for infants while limiting fluid balance, it is recommended to adopt a judicious and personalized method of fluid restriction while meeting the physiological needs of premature infants [43]. Optimal fluid therapy should be titrated to meet normal physiologic and caloric requirements and regulated to achieve a balance between the pulmonary and systemic blood flow [46].

Our study demonstrated that gestational diabetes mellitus and higher Apgar scores were independent risk factors preventing FIT from closing hsPDA. However, we did not find any study showing that gestational diabetes mellitus has an influence on the closure of hsPDA treated with COXI. A recent international cohort study including 78,126 infants also indicated that there was no significant association between gestational diabetes mellitus and hsPDA after adjusting for confounders [50]. Moreover, the independently significant association between higher Apgar scores and a lower rate of successful FIT was contrary to previous studies showing that newborn infants with lower Apgar scores had a higher risk of adverse outcomes [51, 52]. This may be explained by the small sample size and confounding factors not clearly identified in our study.

Through a linear analysis between weight changes and fluid balance, we found that the accuracy of the measured fluid balance in our study was not highly satisfactory. One potential reason may be that we only calculated fluid balance after admission to the NICU but did not take into account fluid balance prior to NICU admission. We also did not analyze insensible water loss and stool when calculating the fluid output volume. Furthermore, examining the accuracy of fluid balance measurements by testing the correlation between weight changes and fluid balance may not be accurate, as changes in infant weight are also affected by other factors, such as protein and calorie intake [53] and related complications, especially severe pulmonary disease [54].

Aside from the accuracy of the measured fluid balance, our study also has other limitations. First, this was a retrospective study and thus was subject to confounding bias with conclusions implying association and not causation. Second, our study had a relatively small sample size of 110 and 27 infants in FIT and SIT, respectively, which limited the power of analyses. Third, this was a single-center study and the generalizability of our findings may not pertain to other centers. Fourth, our unexplained results may be due to the existence of undetected confounders.

## 5. Conclusions

Our data demonstrated that a lower fluid balance volume before FIT, especially within the first week after birth had a significant association with favorable FIT outcome, which might potentially be a therapeutic target to minimize the FIT failure rate. Our study also established the basis for conducting a large randomized controlled clinical trial to examine the effects of restricted fluid administration strategies on the outcome of ibuprofen for hsPDA in preterm infants.

## Data Availability

The datasets used and analyzed during the current study are available from the corresponding author on reasonable request.

## Abbreviations

PDA, patent ductus arteriosus; hsPDA, hemodynamically significant patent ductus arteriosus; FIT, the first course of ibuprofen treatment; SIT, the second course of ibuprofen treatment; IVH, intraventricular hemorrhage; NEC, necrotizing enterocolitis; BPD, bronchopulmonary dysplasia; COXI, cyclooxygenase inhibitors; RDS, respiratory distress syndrome; NICU, neonatal intensive care unit; GA, gestational age; BW, birth weight; SGA, small for gestational age; PROM, premature rupture of membranes; ROC, receiver-operating characteristic; AUC, area under the curve; CI, confidence interval; OR, odds ratio.
